# Ancient origin of a Western Mediterranean radiation of subterranean beetles

**DOI:** 10.1186/1471-2148-10-29

**Published:** 2010-01-28

**Authors:** Ignacio Ribera, Javier Fresneda, Ruxandra Bucur, Ana Izquierdo, Alfried P Vogler, Jose M Salgado, Alexandra Cieslak

**Affiliations:** 1 Museo Nacional de Ciencias Naturales, José Gutiérrez Abascal 2, 28006 Madrid, Spain; 2 Institute of Evolutionary Biology (CSIC-UPF), Passeig Maritim de la Barceloneta 37-49, 08003 Barcelona, Spain; 3 Ca de Massa, E-25526 Llesp, Lleida, Spain; 4 Natural History Museum, Cromwell Road, London SW7 5BD, UK; 5 Imperial College London, Silwood Park Campus, Ascot SL5 7PY, UK; 6 Departamento de Biología Animal, Facultad de Biología, Universidad de León, León, Spain

## Abstract

**Background:**

Cave organisms have been used as models for evolution and biogeography, as their reduced above-ground dispersal produces phylogenetic patterns of area distribution that largely match the geological history of mountain ranges and cave habitats. Most current hypotheses assume that subterranean lineages arose recently from surface dwelling, dispersive close relatives, but for terrestrial organisms there is scant phylogenetic evidence to support this view. We study here with molecular methods the evolutionary history of a highly diverse assemblage of subterranean beetles in the tribe Leptodirini (Coleoptera, Leiodidae, Cholevinae) in the mountain systems of the Western Mediterranean.

**Results:**

Ca. 3.5 KB of sequence information from five mitochondrial and two nuclear gene fragments was obtained for 57 species of Leptodirini and eight outgroups. Phylogenetic analysis was robust to changes in alignment and reconstruction method and revealed strongly supported clades, each of them restricted to a major mountain system in the Iberian peninsula. A molecular clock calibration of the tree using the separation of the Sardinian microplate (at 33 MY) established a rate of 2.0% divergence per MY for five mitochondrial genes (4% for *cox1 *alone) and dated the nodes separating the main subterranean lineages before the Early Oligocene. The colonisation of the Pyrenean chain, by a lineage not closely related to those found elsewhere in the Iberian peninsula, began soon after the subterranean habitat became available in the Early Oligocene, and progressed from the periphery to the centre.

**Conclusions:**

Our results suggest that by the Early-Mid Oligocene the main lineages of Western Mediterranean Leptodirini had developed all modifications to the subterranean life and were already present in the main geographical areas in which they are found today. The origin of the currently recognised genera can be dated to the Late Oligocene-Miocene, and their diversification can thus be traced to Miocene ancestors fully adapted to subterranean life, with no evidence of extinct epigean, less modified lineages. The close correspondence of organismal evolution and geological record confirms them as an important study system for historical biogeography and molecular evolution.

## Background

Isolated or extreme environments, such as islands or high mountains, have been preferred systems for the study of speciation and processes of adaptation [1,2]. One of these "natural laboratories" for evolution is the deep subterranean environment, which combines extreme but homogeneous and constant conditions with a discontinuous distribution promoting isolation [3,4]. Despite the early recognition of the potential value of the subterranean fauna in evolutionary biology (e.g. Darwin devotes three pages of the Origins to discuss the effect of disuse and the convergence among cave species in North America and Europe, [5] pp. 137-139), studies of cave organisms have been hampered by their general scarcity and the difficulty of accessing their habitat.

Most of the evolutionary studies on subterranean fauna have focused on its origin and adaptations [4-6]. Cave or endogean species are characterised by a number of shared characters, assumed to be either caused by loss of function due to "lack of use" (apterism, depigmentation, reduction or complete loss or eyes [5,7]), or adaptations to the harsh environmental conditions in caves or deep soil, such as elongated body and appendages, cold adaptation, modified life cycles, modified fat metabolism, or development of sensory organs [3,6,8] (see [9-11] for specific examples of Pyrenean Leptodirini). Subterranean species are usually considered "super specialists", which cannot survive outside the narrow range of highly stable conditions found in their habitats, and have very limited potential to disperse [4]. As a result, the geographical range of subterranean species is usually very restricted, in many cases to a single cave or a karstic system [8,12]. Species traditionally considered to have wide distributions had often been shown to be complexes composed of multiple cryptic lineages when molecular methods were applied (e.g. [13,14]).

A widely accepted view on lineage evolution in cave organisms is that subterranean species are evolutionary dead-ends that do not disperse once fully adapted to the environment of deep caves or soil. They are prone to become extinct before being replaced by lineages newly derived from epigean species that re-colonise the subterranean medium [4,15]. There are two general hypotheses regarding the origin of the subterranean fauna (see [4,16,17] for reviews): the climatic relict and the habitat shift hypotheses. Briefly, under the climatic relict hypothesis [18] the subterranean medium acts as a refuge for epigean fauna in times of unfavourable climatic conditions. The populations that are forced into a subterranean habitat become isolated from their epigean relatives and eventually develop the morphological and physiological adaptations to this new environment. In lineages with exclusively subterranean species the lack of close epigean relatives is due to extinction of the latter, leaving the subterranean species as the only survivors that escaped extinction [18]. The adaptive shift hypothesis [19,20] surmises that the colonisation of the subterranean medium is driven by the opportunity to exploit new resources. Epigean populations are not forced below ground by changing conditions, and there may be limited genetic flux between the two environments for some time. Both scenarios assume multiple origins of the subterranean lineages from closely related epigean relatives, with recurrent colonisation of the subterranean medium.

There is, however, little phylogenetic evidence to support these hypotheses, especially for the most diverse lineages of exclusively subterranean species, such as some groups of beetles. Among Coleoptera there are multiple examples of phylogenetically independent cave or endogean groups, in particular in the families Carabidae, Staphylinidae and Leiodidae [21,22]. Many of these are either species poor subterranean lineages or a mix of hypogean and epigean species, with different degrees of morphological modifications and ecological specialisation. Only a few groups appear to represent extensive monophyletic radiations of exclusively subterranean species. Among them, the tribe Leptodirini in the family Leiodidae [21] includes at present ca. 240 recognised genera and ca. 1,800 species [23], which are almost exclusively subterranean. The highest diversity is found in the Mediterranean basin, in particular in the north and east of the Iberian peninsula, some Mediterranean islands (Corsica and Sardinia), the southern Alps, Balkan peninsula, Romania and southern Russia, the Caucasus, Middle East and Iran [23]. The few Leptodirini found east of Iran, and the only two Nearctic species (in the genus *Platycholeus*), are of uncertain phylogenetic affinities ([24,25], see Discussion).

**Figure 1:**
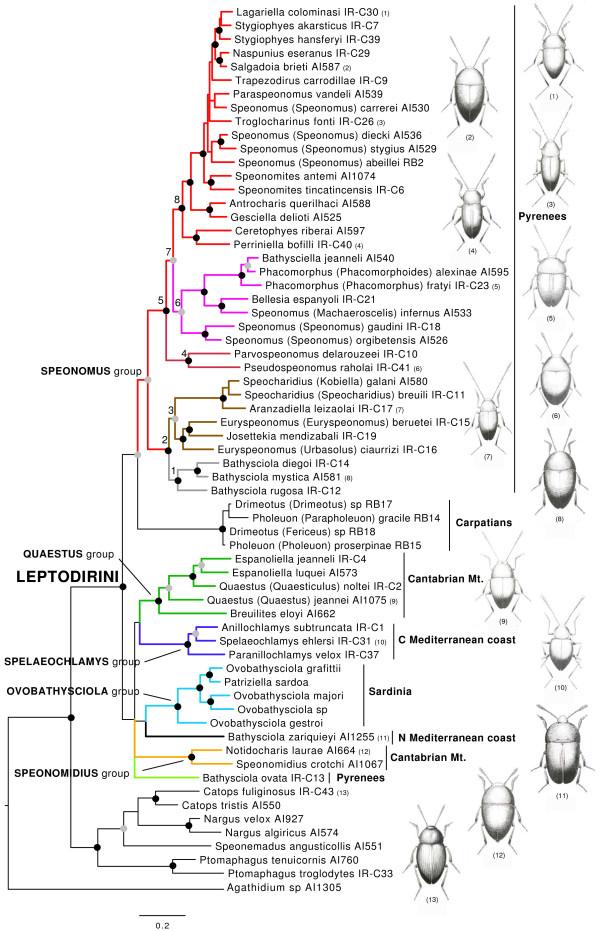
**Phylogram obtained with MrBayes with the MAFFT alignment including the Sardinian species, with the geographical area in which the main lineages are distributed (see Fig. 2)**. Black circles, well supported nodes (Bayesian pp >0.95, ML bootstrap >70%) for the four measures (Bayesian in MrBayes, ML in Garli, each for the MAFFT and PRANK alignments). Grey circles, good support in at least two measures, the others at least bootstrap >50% or pp >0.5. See Additional file 3 for the detailed values of support, and Fig. 2 for the distribution of the main clades. Habitus of species, from top to down: (1) *Lagariella colominasi *(Zariquiey), (2) *Salgadoia brieti *(Jeannel), (3) *Troglocharinus kiesenwetteri *(Dieck), (4) *Perriniella faurai *Jeannel, (5) *Phacomorphus fratyi *(Dupré), (6) *Pseudospeonomus raholai *(Zariquiey), (7) *Aranzadiella leizaolai *Español, (8) *Bathysciola mystica *Fresneda & Fery, (9) *Quaestus arcanus *Schauffus, (10) *Spelaeochlamys ehlersi *Dieck, (11) *Bathysciola zariquieyi *Bolívar, (12) *Notidocharis uhagoni *(Sharp), (13) *Catops nigricans *(Spence).

The monophyly of the western Palaearctic Leptodirini is strongly supported by morphological characters [24-26], but their internal phylogeny was only recently addressed with numerical methods, and mostly with morphological analysis of the internal structure of the male genitalia, as other external morphological characters show extreme homoplasy [25] (see Fig. 1 for an overview of the morphological diversity of the group). The resolution and support attained with these character sets is, however, very limited, and was unable to provide a robust phylogenetic framework.

We provide here a comprehensive molecular data set for the main lineages of Leptodirini present in the Western Mediterranean, including the Iberian peninsula (plus adjacent mountain massifs in Southern France) and Sardinia. The known fauna of this region includes ca. 40 genera with some 230 mostly obligatory troglobiontic species, but also some endogean or muscicolous species in the genera *Bathysciola *and *Notidocharis *[23] (Additional file 1). We aim to establish a robust phylogeny to study the evolution of this extensive subterranean species radiation, and to provide a temporal framework for the diversification of various lineages and the colonisation of the geographical areas in which they occur.

## Methods

### Taxon sampling

We follow the classification of Lawrence & Newton [27] and Perreau [23] down to tribe level. All ingroup taxa in the study are currently included in Leiodidae: Cholevinae: Leptodirini, although the formal classification of Leptodirini is in need of a phylogenetic revision [25]. We use here "group of genera" (or "group" for simplicity) for lineages found to be monophyletic in our analyses, to avoid confusion with the traditionally defined "series" which in the French school of mid-20^th ^century entomology did not necessarily imply monophyly in its modern sense [28]. We sampled representatives of all major lineages present in the Western Mediterranean (31 out of the 42 genera occurring in the Iberian peninsula), plus the two endemic Sardinian genera using data from [29] and two genera from the Carpathians as examples of Eastern lineages, *Drimeotus *and *Pholeuon *[23] (Additional file 1). No Leptodirini are known from the Balearic islands. Sardinia has three additional species currently included in *Speonomus *subgenus *Batinoscelis *and four species of *Bathysciola*, and Corsica five species of *Parabathyscia*, a genus of the *Bathysciola *series *sensu *Perreau [23] with Alpine and Eastern European affinities. Most Western Mediterranean missing genera could readily be associated to clades of the sampled taxa according to the structure of the aedeagus, which we show here to be a character with reliable phylogenetic information (see [25,30] for a detailed taxonomic discussion).

The final data matrix included 57 species of Leptodirini and 8 outgroups from different tribes of Cholevinae (Anemadini, Ptomaphagini, Cholevini; Additional file 1) and *Agathidium *as a member of the phylogenetically separated subfamily Leiodinae [26,31].

### DNA extraction, amplification and sequencing

The specimens used in the study were preserved in absolute ethanol in the field. A full list of collectors and localities is given in Additional file 1. Extractions of single specimens were non-destructive, using a standard phenol-chloroform method or the DNeasy Tissue Kit (Qiagen GmbH, Hilden, Germany). Vouchers and DNA samples are kept in the collections of the Natural History Museum, London (NHM) and Museo Nacional de Ciencias Naturales, Madrid (MNCN) (Additional file 1).

We amplified fragments of seven genes, five mitochondrial and two nuclear: 3' end of cytochrome c oxidase subunit (*cox1*); 5' end of the large ribosomal unit plus the Leucine transfer plus the 3' end of NADH dehydrogenase subunit 1 (*rrnl*+*trnL*+*nad1*); an internal fragment of cytochrome b (*cob*); 5' end of the small ribosomal unit, 18S rRNA (*SSU*); and an internal fragment of the large ribosomal unit, 28S rRNA (*LSU*). Primers used are given in Table 1, and PCR protocols are given in [32,33]. Sequences were assembled and edited using Sequencher TM 4.1.4 (Gene Codes, Inc., Ann Arbor, MI). New sequences have been deposited in GenBank (NCBI) with Acc. Nos GU356744-GU356993 (Additional file 1). In two cases (*Quaestus noltei *(Coiffait) and *Bathysciola ovata *(Kiesenwetter)) the final sequence was a composite of two different specimens of the same species (Additional file 1).

**Table 1:** Primers used in the study. F, forward; R, reverse.

Gene	Name	Sense	Sequence	Reference
*cox1*	Jerry (M202)	F	CAACATTTATTTTGATTTTTTGG	[72]
	Pat (M70)	R	TCCA(A)TGCACTAATCTGCCATATTA	[72]
	Chy	F	T(A/T)GTAGCCCA(T/C)TTTCATTA(T/C)GT	A. Cieslak, this work
	Tom	R	AC(A/G)TAATGAAA(A/G)TGGGCTAC(T/A)A	A. Cieslak, this work
	Tom-2	R	A(A/G)GGGAATCATTGAATAAA(A/T)CC	A. Cieslak, this work
*cob*	CB3	F	GAGGAGCAACTGTAATTACTAA	[73]
	CB4	R	AAAAGAAA(AG)TATCATTCAGGTTGAAT	[73]
*rrnL*-*nad1*	16saR (M14)	F	CGCCTGTTTA(A/T)CAAAAACAT	[72]
	16Sa	R	ATGTTTTTGTTAAACAGGCG	[72]
	16Sb	R	CCGGTCTGAACTCAGATCATGT	[72]
	16SAlf1	R	GCATCACAAAAAGGCTGAGG	[74]
	ND1A (M223)	R	GGTCCCTTACGAATTTGAATATATCCT	[72]
	16Sbi	F	ACATGATCTGAGTTCAAACCGG	[72]
	FawND1	R	TAGAATTAGAAGATCAACCAGC	[72]
*SSU*	5'	F	GACAACCTGGTTGATCCTGCCAGT	[32]
	b5.0	R	TAACCGCAACAACTTTAAT	[32]
*LSU*	Ka	F	ACACGGACCAAGGAGTCTAGCATG	NHM (2000)
	Kb	R	CGTCCTGCTGTCTTAAGTTAC	NHM (2000)

No specimens from Sardinia were available for sequencing, but sequences of the same fragment of the *cox1 *gene used here (776 overlapping positions) could be obtained for several Sardinian species from GenBank [29]. We excluded sequences reported to be obtained from dry material in [29], as they were placed in unlikely positions in the phylogeny or had large numbers of autapomorphies,

### Phylogenetic analyses

We used two different approaches for multiple progressive pair-wise alignment, either with secondary refinement using the MAFFT online v.6 and the Q-INS-i algorithm [34] ("MF" in the following) or with modelling the evolution of indels with PRANK [35] ("PR").

Bayesian analyses were conducted on a combined data matrix with MrBayes 3.1.2 [36], using six partitions corresponding to the six genes, but combining the short *trnL *together with the *rrnL *gene. Evolutionary models were estimated prior to the analysis with ModelTest 3.7 [37]. MrBayes ran for 5 × 10^6 generations using default values, saving trees at each 500^th ^generation. "Burn-in" values were established after visual examination of a plot of the standard deviation of the split frequencies between two simultaneous runs.

We also conducted maximum likelihood searches in Garli v.0.951 http://www.bio.utexas.edu/faculty/antisense/garli/Garli.html, which uses a stochastic genetic algorithm-like approach to simultaneously find the topology, branch lengths and substitution model parameters [38]. We used an estimated GTR+I+Γ model for the combined sequence (as estimated with ModelTest), and the default settings. Support was measured with 1,000 bootstrap replicates, reducing the number of generations without improving the topology necessary to complete each replicate to 5,000.

### Estimation of molecular rates and dates of diversification

For the age calibration of the phylogenetic tree we used the point of separation between the Sardinian and the continental lineages. The Sardinian clade most likely originated by allopatric separation when the Sardinian microplates split from continental Europe through tectonic movements [29]. Recent tectonic reconstructions [39,40] allow both a detailed geographical analysis of the relative positions of the microplates to the continent and fixing a minimum date for the origin of the Sardinian clade. The tectonic movements leading to the separation of the Western Mediterranean microplates started not before 33 MY ago, and by 25 MY ago the separation from Iberia and the Gulf of Lyon was complete, although some connections with the Italian peninsula may have persisted [40]. However, given the limited dispersal among subterranean populations the geological uncertainty as to the existence of remnant land bridges are likely to be unimportant for the dating of the separation of Leptodirini. The use of a hard bound minimum age calibration of 25 MY as the latest possible population split would bias the estimations towards younger ages [41], so we use the earlier date of 33 MY as the most plausible time of separation between the Sardinian clade and its sister, which may still be conservative (too recent) if the separation of the lineages predated the split of the microplates.

Because available sequences for the species of the Sardinian clade were limited to the *cox1 *marker (see above) we conducted the Bayesian tree searches including or excluding the sequences of the Sardinian species, to test for the effect of missing data. The topologies of the resulting trees were identical (see Results). We therefore used the topology obtained with the Bayesian analyses for the whole dataset with the PR alignment (which was fully compatible with that obtained with MF, but more resolved; see below), to apply two methods to obtain an ultrametric tree: Bayesian estimations, as implemented in Beast 1.4.7 [42], and penalized likelihood [43], as implemented in r8s 1.71 http://ginger.ucdavis.edu/r8s/. We used the branch lengths corresponding to the single *cox1 *sequence (pruning the species with missing *cox1 *from the tree), with a GTR+I+Γ model with parameters estimated in PAUP [44].

For the Bayesian analyses in Beast, well supported nodes in the analyses with the combined mitochondrial and nuclear sequence were constrained to be monophyletic (see Results), and a GTR+I+Γ model was enforced with an uncorrelated lognormal relaxed clock and a Yule diversification model [42]. All parameters were set to default values, with the exception of the prior of the age of the clade formed by the Sardinian species and their sister, which was set to a normal distribution with mean of 33 MY and a standard deviation of 2.0 MY (corresponding to a 95% confidence interval of 28.8 and 36.3 MY) to allow for variance from stochastic sampling error of nucleotide changes [41]. The results of two independent runs were merged with Tracer v1.4 and TreeAnnotator v1.4.7 [42].

In r8s we used the Truncated Newton algorithm and performed a cross-validation procedure with smoothing factors between 1 and 500 (after elimination of the outgroups) [43]. Once the optimal smoothing factor was found, the program was run again using 33 MY as a minimum constraint for the node including the Sardinian clade and its sister. A confidence interval of the rates was estimated using the profiling option in r8s with the topologies of the last 1,000 trees in one of the MrBayes runs, with branch lengths for *cox1 *estimated in PAUP as above. Note that because the branch lengths are not those originally estimated in MrBayes for the whole combined sequence, but those estimated in PAUP for *cox1 *alone, the stochastic variation reflected in the confidence interval is the result of the different topologies of the 1,000 trees used, plus the variation introduced subsequently by rate smoothing in r8s.

To test for possible artefacts due to the inclusion of only the *cox1 *sequence, and to obtain a rate calibration for other mitochondrial genes, we performed additional analyses in Beast including all the mitochondrial sequences but excluding the Sardinian species. The node below that of the insertion of the Sardinian species was constrained to have the age estimated with *cox1 *alone, with a normal distribution with standard deviation of 2.0 MY. Beast was run for 5 × 10^6 generations, saving trees at every 500th generation.

## Results

### Phylogeny of Western Mediterranean Leptodirini

The protein-coding genes (*cox1, cob, nad1*) had no indels, with the single exception of a three to four nucleotide insertion beyond the end of the *cox1 *gene (in the region of the *trnL*) in the *Quaestus *group of genera and in *Aranzadiella*. Most of the length variation was concentrated in the hypervariable regions of the *LSU *fragment, while for the *rrnL *and *SSU *genes the length variation between the two alignment methods used (MF and PR) was relatively small (Table 2). For the *LSU *gene the PR alignment was more than 1,000 positions longer than the MF alignment, with a loss of 79 informative characters (Table 2). Despite the large differences in the length of the combined sequence between the two alignment methods (mostly due to *LSU*), the changes in the topology or level of support of the resulting trees were negligible (see below).

**Table 2:** Length of the sequenced fragments, with maximum and minimum length and number of nucleotides in the matrix (No.) and number of informative characters (Inf.) in the two alignments.

	Raw length		MAFFT		PRANK	
	
gene	min	max	No.	Inf.	No.	Inf.
*cox1*	826	830	830	380	830	380
*rrnL*+*trnL*	668	705	727	248	756	257
*nad1*	378	378	378	176	378	176
*cob*	358	358	358	176	358	176
*SSU*	591	606	647	69	612	56
*LSU*	579	774	923	252	2141	173

The optimal evolutionary model was GTR+I+Γ, both for the combined data and the individual gene fragments. The two independent Bayesian runs reached values of the standard deviation of the split frequencies between them of ca. 0.005 for both the PR and MF alignments. The estimated parameters for the two alignments were within the 95% confidence intervals of each other, with the single exception of alpha (α) of the *SSU *and *LSU *partitions, and the probability of invariant sites (I) of the *LSU *partition (Additional file 2). The topologies of the trees obtained with Bayesian probabilities for the two alignments and those obtained with ML in Garli were either identical or compatible with each other (i.e. consensus consistent with resolved nodes). The differences in tree topology only affected the relationships of one of the outgroups (*Speonemadus angusticollis *Kraatz) and the internal phylogenetic relationships of the Carpathian clade (Fig. 1, Additional file 3). Nodal support was generally high, with most posterior probability values >0.95 and maximum likelihood boostratp values >70% (Additional files 3, 4), with generally higher support values at some deeper nodes with Bayesian methods (Additional file 3), as had been observed in other studies [45].

The exclusion of the Sardinian species (due to missing data, see Methods) in a Bayesian analysis using the MF alignment produced a topology identical to that obtained with all species (Additional file 4).

**Figure 2:**
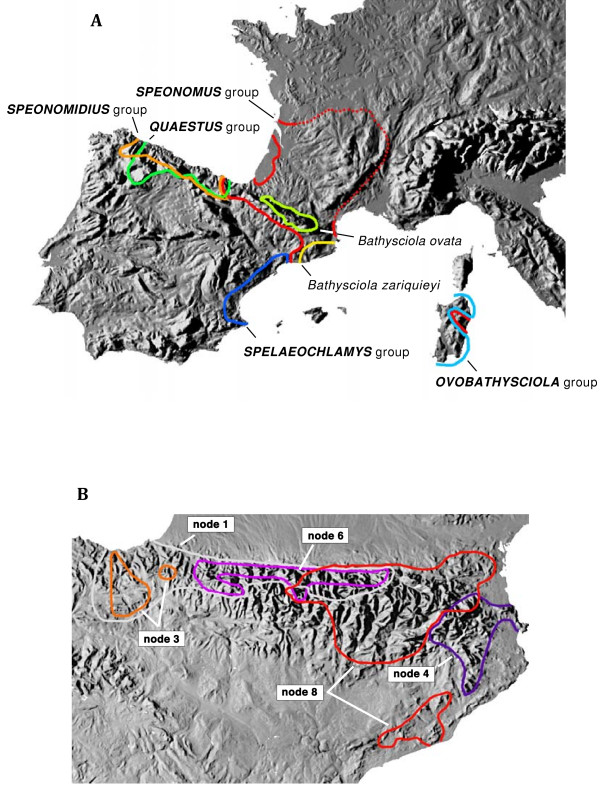
**Distribution of the main lineages of W Mediterranean Leptodirini as established in this study**. (a), General distribution of the main lineages (see Fig. 1); (b) detailed distribution of the *Speonomus *group of genera in the Pyrenees (see Fig. 1).

All species of Leptodirini formed a well supported clade, to the exclusion of other tribes of Cholevinae (Fig. 1). Within the Leptodirini, well supported major lineages were composed of species restricted to particular biogeographical regions (Figs 1, 2). In most cases the main lineages were fully allopatric, as separate clades were confined to the Pyrenees (*Speonomus *group of genera), the Cantabrian Mountains (*Quaestus *and *Speonomidius *groups), the central Mediterranean coast of Iberia (*Spelaeochlamys *group), Sardinia (*Ovobathysciola *group), and the Carpathians. In addition, two species of *Bathysciola*, *B. zariquieyi *Bolívar distributed throughout the coastal mountain systems in Catalonia [46] and *B. ovata*, with a wide distribution in Pyrenees and SE France, constitute phylogenetically isolated lineages separated from other members of the genus (Figs 1, 2).

The monophyletic Pyrenean lineage, corresponding to the *Speonomus *series of Fresneda *et al*. [25], was sister to the genera from the Carpathians (Fig. 1), and was split in two main lineages defined by nodes 2 and 5 in Fig. 1. The former includes genera from the lower western areas (node 3, Basque Country and Navarra) plus some endogean and muscicolous *Bathysciola *with a wider distribution towards the east. The clade defined by node 5 includes genera from the eastern lowland areas (node 4, *Parvospeonomus *and *Pseudospeonomus*) plus a large clade of genera from the central Pyrenees (node 7). The latter clade was also geographically structured, with genera roughly distributed west (node 6) and east (node 8) of the river Gállego in the central Pyrenees (Figs 1, 2). The genus *Troglocharinus *(included in node 8) has a disjunct distribution, with some species distributed in the southern coastal ranges of Catalonia (Fig. 2b; [30]), although the monophyly of the genus could not be tested as the southern species were missing from our study.

The remaining Leptodirini consisted of several highly supported clades with very well defined distributions: (1) the *Quaestus *group in the Cantabrian mountains; (2) the *Spelaeochlamys *group in the Mediterranean coast of Spain; (3) the *Ovobathysciola *group in Sardinia; (4) the divergent species *Bathysciola zariquieyi *from the Catalonian mountains; (5) the *Speonomidius *group of Cantabria; and (6) *Bathysciola ovata *from the Pyrenees (Figs 1, 2a). The relationships among these lineages were in general poorly resolved, although the sister relationship between the *Quaestus *and *Spelaeochlamys *groups, and of *Bathysciola zariquieyi *and the Sardinian clade, were well supported in some Bayesian analyses (Additional file 3).

### **Temporal diversification of the Western Mediterranean Leptodirini**

**Figure 3:**
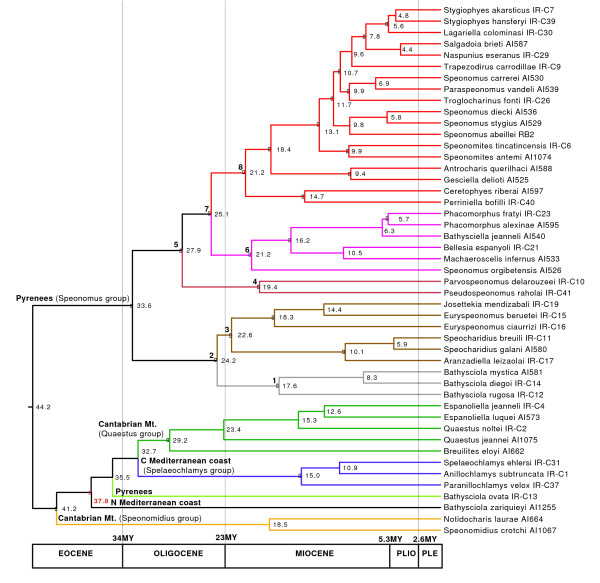
**Ultrametric tree obtained with Beast using the combined sequence, excluding the Sardinian species**. Black circles, well supported nodes (see Fig. 1, Additional file 3) constrained to be monophyletic. Numbers inside nodes, age estimate (MY), using the separation of *Bathysciola zariquieyi *from its sister (red node), with a prior age of 37.9MY (see text and Additional file 5).

The split between the Sardinian clade and its mainland sister *Bathysciola zariquieyi *was used for estimating the evolutionary rate of *cox1*, using a calibration point of 33 MY ago as the time of vicariant separation of both lineages (Fig. 1). When using Beast, the rate estimate was 0.020 +/- 0.005 substitutions per site per MY, i.e. an overall pair-wise divergence of 4.0% per MY (Table 3, Additional file 5). The node that grouped *B. zariquieyi *plus the Sardinian clade with their sisters was estimated to date back to ca. 38 +/- 7 MY ago. This constraint was subsequently used for the estimation of the evolutionary rate for the combined mitochondrial genes (excluding the species of the Sardinian clade for which these data were missing). The rate of the five combined mitochondrial markers (*cox1*, *rrnL*, *trnL*, *nad1 *and *cob*, comprising 2,293 bp in the MF alignment; Table 2) was 0.010 +/- 0.002, equivalent to an overall pair wise divergence of 2.0% per MY (Table 3). The estimation of node ages using the combined mitochondrial markers was very similar to that obtained using *cox1 *only, and fully within the 95% confidence interval of each other (Table 3, Fig. 3, Additional files 3, 6).

**Table 3:** Estimated rates of molecular evolution and ages of the constricted nodes, with confidence intervals.

		Beast, mitochondrial			Beast, *cox1*			r8s, *cox1*		
		
		mean	lower	upper	mean	lower	upper	mean	lower	upper
	Rate	0.0099	0.0081	0.0116	0.0204	0.0151	0.0260	0.0115	0.0113	0.0117
1	root	44.16	37.46	50.76	45.41	35.87	56.03	39.62	.	.
2	**Sardinian clade [*cox1*]**	n.a.	n.a.	n.a.	**32.75**	**28.96**	**36.76**	**33.00**	.	.
3	**Sardinian clade [mt]**	**37.79**	**33.86**	**41.84**	37.92	31.43	45.43	36.92	.	.
4	infraflagellates	41.18	35.60	46.82	41.42	33.04	50.28	36.92	20.69	39.09
5	*Speonomus *group	33.61	27.67	39.82	36.32	27.53	46.23	30.45	22.81	32.50
6	*Speonomidius *group	18.51	13.70	23.69	22.67	14.37	31.31	20.70	.	.
7	*Spelaeochlamys *group	15.04	11.31	18.98	13.63	9.08	18.32	15.97	.	.
8	*Quaestus *group	29.20	24.06	34.37	30.54	23.03	37.94	31.11	.	.
9	node 1	17.59	13.39	22.12	17.56	11.00	24.23	18.02	.	.
10	node 2	24.16	19.47	29.12	23.59	17.29	30.58	21.88	.	.
11	node 3	22.59	18.03	27.23	22.41	16.46	29.04	21.88	.	.
12	node 4	19.40	14.64	24.47	22.34	14.69	30.32	20.80	.	.
13	node 5	27.94	22.95	33.34	31.46	23.39	39.90	26.94	.	.
14	node 6	21.18	17.06	25.63	23.29	17.00	29.94	21.24	.	.
15	node 7	25.08	20.71	29.89	26.95	20.28	34.18	25.08	.	.
16	node 8	21.22	17.28	25.37	21.44	16.06	27.29	19.63	.	.
17	*Aranzadiella*+*Speocharidius*	10.09	7.39	12.89	8.99	5.81	12.52	9.85	.	.
18	*Euryspeonomus*+*Josettekia*	18.27	14.20	22.89	18.07	12.62	24.29	18.92	.	.
19	*Phacomorphus*-*Bellesia*	16.18	12.76	19.75	16.70	11.83	21.73	15.84	.	.
20	*Phacomorphus*	5.70	4.11	7.37	5.71	3.63	7.88	4.83	.	.
21	*Perriniella*+*Ceretophyes*	14.72	10.86	18.65	17.93	12.00	24.12	17.37	.	.
22	*Gesciella*-*Antrocharis*	9.45	6.56	12.42	12.11	7.32	17.08	13.72	.	.
23	*Speonomites*	9.96	7.36	12.65	9.61	6.25	13.16	9.49	.	.
24	*Gesciella*-*Stygiophyes*	18.38	14.92	22.17	19.74	14.69	25.36	18.95	.	.
25	*Speonomites*-*Stygiophyes*	13.06	10.55	15.63	12.48	9.28	15.96	12.26	.	.
26	*Stygiophyes*-*Speonomus*	11.71	9.52	14.04	11.21	8.32	14.32	11.67	.	.
27	*Speonomus*	9.85	7.68	12.11	9.69	6.96	12.67	10.37	.	.
28	*Stygiophyes*-*Troglocharinus*	10.70	8.67	12.83	9.76	7.14	12.49	10.59	.	.
29	*Stygiophyes*-*Salgadoia*	7.85	6.18	9.59	7.67	5.46	10.11	8.61	.	.
30	*Stygiophyes*+*Lagariella*	5.63	4.32	7.10	5.55	3.70	7.48	6.48	.	.

See Fig. 3 & Additional file 5 for the definition of the nodes. In bold, nodes used for the calibration (see Methods).

For comparison we estimated the age of the nodes with the Penalized Likelihood (PL) method in r8s [43], using *cox1 *only and a calibration date of 33 MY for the Sardinian-continental split. Estimated node ages throughout were very similar to those obtained with Bayesian methods, and well within their 95% confidence interval, with the sole exception of the node defining the sister relationship between *Antrocharis *and *Gesciella *(older with the PL estimation; Table 3 and Additional file 6). However, the estimated overall rate was 0.0115 +/- 0.0005 (2.3% per MY, Table 3), i.e. lower than the estimate obtained with Beast for the *cox1 *gene, suggesting substantial differences in the branch length estimates from both methods.

The main lineages of Western Mediterranean Leptodirini were estimated to have diversified between ca. 45-33 MY, during the Mid to Late Eocene (Fig. 3). The relative age of the nodes suggests that by the time the Sardinian lineage formed (presumably by Late Eocene - Early Oligocene) all other main lineages were already present and distributed over their present geographical areas. All lineages with a well defined geographical distribution were estimated to have originated by the Late Oligocene (Figs 2, 3). The age of diversification within the genera for which more than one species was sequenced ranged from ca. 20 to 5 MY (mostly Miocene), disregarding some cases where the currently recognised genera were found to be paraphyletic (*Euryspeonomus*, *Quaestus*) or polyphyletic (*Speonomus*, *Bathysciola*) (Fig. 1).

## Discussion

The most prominent feature of the phylogeny of the Western Mediterranean Leptodirini is the strong geographical division among the main lineages. Support for these geographically restricted clades is generally very strong and stable to changes in alignment or reconstruction methods, while the relationships among them is less well supported. All well supported deep clades were restricted to a single mountain massif, and each of these is occupied by a single clade with the sole exceptions of the Cantabrian mountains, with two separate lineages (the *Quaestus *and *Speonomidius *groups of genera) and some species of *Bathysciola *in the Pyrenees. In both cases one of these sympatric lineages is composed of cave species only (*Quaestus *and *Speonomus *groups) while the other is muscicolous or endogean (*Speonomidius *and *Bathysciola *for the Cantabrian mountains and the Pyrenees respectively) (see Additional file 1 for the habitat of the studied species). It would be interesting to compare the rates of diversification of the different lineages in the same geographical area, and the occurrence of species in sympatry to test for the possible effect of competitive exclusion within each habitat type.

### **Phylogeny of Western Mediterranean Leptodirini**

Our results basically agree with the traditional taxonomic division of Leptodirini in "infraflagellates" [25] and "supraflagellates" [8,28], based on the complex structure that is characteristic of the aedeagus in the former (the "Y piece" [25]). In our study the infraflagellates correspond to the Pyrenean and the Carpathian species, albeit supported only in the Bayesian inference and with the exclusion of some species of *Bathysciola*. The polyphyly of the genus *Bathysciola *has already been established (see [25]), but the inclusion of some species among the "supraflagellates" implies multiple independent origins of a complex aedeagus. Alternatively, the presence of the "Y piece" could be the ancestral condition of Leptodirini, which underwent repeated simplifications in at least the Cantabrian and Sardinian species of the *Ovobathysciola *group (Fig. 1), contrary to expectations (e.g. [8,26,28]).

Our results also support most of the traditional "phyletic series" of Jeannel [8,28], while in Fresneda *et al*.'s [25] study using characters of the external morphology and the internal genitalia, support was found only for the monophyly of the *Speonomus *(including *Bathysciola*) and *Spelaeochlamys *plus *Ovobathysciola *series. The molecular analyses also supported the traditionally defined *Speonomidius*, *Quaestus *and *Spelaeochlamys *series [30,47-49]. The *Speonomidius *series includes the genus *Notidocharis *[47], with seven muscicolous rather than subterranean species, all with reduced eyes (Additional file 1). Other muscicolous oculated Leptodirini include some *Bathysciola*-like taxa in Anatolia, the Caucasus and north Iran, plus *Adelopsella bosnica *(Reitter) from the Balkans and two Nearctic species in the genus *Platycholeus *[50]. The genera *Adelopsella*, *Platycholeus *and *Sciaphyes *(tribe Sciaphyini from east Siberia [23]) share some unique characters not present in the remaining Leptodirini [8], which suggests they may not be closely related. According to Fresneda *et al. *[25] the presence of eyes placed *Notidocharis *as sister to the rest of studied Leptodirini. In the current analysis this position is not strongly contradicted, as the support of the basal nodes of Leptodirini in the trees obtained with ML was very weak (Fig. 1, Additional file 3).

The Pyrenean clade corresponds to the *Speonomus *series as defined by Fresneda *et al*. [25] with the exclusion of some species of *Bathysciola*, but confirming the inclusion of the genus *Pseudospeonomus *(= *Pseudochlamys *Comas), distributed in the extreme east of the Pyrenees and previously grouped with species in the mountains of the Mediterranean coast of Spain [25]. The Sardinian species of the *Ovobathysciola *group, although grouped with the Mediterranean species (*Spelaeochlamys *group) in studies of morphology [25,29], seem more closely related to *Bathysciola zariquieyi *according to the DNA data. The support for this relationship was low under ML, possibly due to missing data (only the *cox1 *sequence was available for the Sardinian clade [29]), but reasonably high with Bayesian methods (pp > 0.9 for both the MF and PR alignments). *Bathysciola zariquieyi *has a relatively wide distribution in the Catalonian coast, from south of Barcelona to the province of Girona [46]. The Catalonia-Sardinia link is more in agreement with geological reconstructions [40], according to which the Sardinian microplates were last connected to the mainland in the north (French coast), rather than the south Iberian Mediterranean coast, as previously assumed [29].

According to our results the Leptodirini fauna of the Iberian peninsula is most likely not monophyletic. The Pyrenean clade seems most closely related to some Eastern European lineages, and the lineage of the Mediterranean coast (*Spelaeochlamys *group of genera) is related to the Sardinian *Ovobathysciola *group and to some species north of the Ebro basin and in southern France.

### Calibration

The use of molecular data to estimate the ages of extant taxa has revolutionised many aspects of evolutionary biology [51,52], despite their known limitations and shortcomings [51,53]. There is, however, a great scarcity of good reference calibrations for many groups, and the "standard" arthropod mitochondrial rate of 2.3% per MY [54] is often used uncritically for a variety of genes, ages and organisms. Subterranean species have been used to calibrate molecular phylogenies, as their distributions presumably are more likely to reflect ancient vicariant events due to their low dispersal (e.g. [29]). However, these ancient vicariant scenarios could be confounded by occasional dispersal of minute endogean species, or in lineages where above-ground dispersal of epigean forms preceded the shift to the subterranean environment. Our calibrations depend on the vicariance between the Sardinian and the European plates, i.e. the presence of a common ancestor in this region prior to the split of these landmasses. Strictly, we cannot exclude an independent origin of subterranean habits in the Sardinian *Ovobathysciola *group, or drifting of a *Bathysciola*-like endogean ancestor to the island subsequent to its separation from the continent, but these seem to be unlikely scenarios. Using this calibration point, the average rate for the sequenced mitochondrial genes (*cox1*, *cyb*, *rrnL *and *nad1*) was 2% per MY, surprisingly close to the standard 2.3% [54], and in agreement with results from other calibrations for beetles [12,55-57]. The *cox1 *gene alone produces a twofold faster rate (4%) compared to the averaged mitochondrial rate including the slower *rrnL *and *trnL *genes, also in agreement with previous results (e.g. [58,59]). The age estimations using Beast and r8s, whether using *cox1 *or the combination of several protein coding and ribosomal mitochondrial genes, were remarkably similar (Table 3, Additional file 6), despite differences in the estimation of the rate for the *cox1 *gene between r8s and Beast. As noted above, this may be related to the differences in the method used for the computation of the branch lengths (Bayesian methods in Beast, penalized likelihood in r8s [42,43]).

### Evolution of the Western Mediterranean Leptodirini

The estimations of node ages date the origin of the main lineages of Leptodirini to the Early Eocene, with the initial diversification taking place during the Oligocene (ca. 35-20 MY, Fig. 3). Our sampling does not include some lineages of Leptodirini including genera from the Alps, Balkans or mainland Italy, but these are mostly "infraflagellates", [26,28], i.e. likely to be embedded in the existing clades. Due to the finding of the Carpathian as sister to the Pyrenean lineage, and the inclusion in our dataset of the oculated, muscicolous *Notidocharis*, it is unlikely that these missing lineages could move the origin of the lineages further back in time.

The diversification of the main lineages during the Paleogene coincides with the closure of the Tethys Sea and the collision of the Iberian and Eurasian plates [39]. Under this scenario, the origin of the Pyrenean lineage of Leptodirini at 34 +/- 6 MY ago followed shortly upon the formation of the Pyrenees during the Alpine orogeny which was largely completed by the Early Oligocene [60,61]. The colonisation of the Pyrenean subterranean medium therefore took place without delay after suitable habitat was available, i.e. after the karstification by water erosion of the recently raised calcareous massifs. This view is strengthened by the fact that the basal cladogenetic splits involve the species at both edges of the chain (nodes 2 and 4 in Fig. 3, see Fig. 2b), with subsequent splits including species more centrally distributed (node 6, plus *Ceretophyes *and *Perriniella*), and the species in the central part, with the highest elevations, among the most recently derived lineages (node 8). This centripetal early colonisation, completed ca. 10 +/- 3 MY ago (Fig. 3), was followed in some cases by an intra-genus diversification in each of the valleys or mountain massifs to which these groups are currently confined. The origin of the main lineages of Leptodirini in the Cantabrian mountains and the Mediterranean coast appears slightly older than in the Pyrenees. This is in agreement with a likely older age of the available habitat, as these mountain areas were present in the Iberian plate before the formation of the Pyrenees [39]. The Oligocene to mid Miocene origin of the main lineages (above what is currently recognized at the level of genus) is older than for other lineages of subterranean European fauna whose origin was placed in the Late Miocene to Pleistocene ([62,63] and references therein), including the Pyrenean radiation of subterranean Trechini groundbeetles [22].

The strong geographical structure of subterranean taxa at multiple hierarchical levels is a common pattern also evident in other recent studies (see e.g. [64,65] for Crustacea; or [12,22] for Coleoptera). Each well defined biogeographical unit is occupied by a single monophyletic lineage, while any phylogenetic substructure within such groups frequently reflects the geographical subdivision of these wider areas, in many cases contrary to expectations from morphological similarities. In some cases these geographically confined lineages include both subterranean and epigean species, e.g. stygobiontic Crustacea [66,67], spiders and beetles in the tribe Trechini in the Canary Islands [56,68], or stygobiontic diving beetles in Australia [12]. These examples provide strong evidence for multiple colonisation of the subterranean medium, as they include lineages that exhibit different degrees of morphological modifications. Their adaptations to the subterranean environment is in accordance with the adaptive shift hypothesis [19], that invokes multiple independent colonisations of the subterranean medium and continued survival of the epigean ancestors (see Introduction). In the Western Mediterranean Leptodirini, the only lineage with non-subterranean, oculated muscicolous species (*Notidocharis*) is placed in an ambiguous position close to the origin of the western Mediterranean lineages, i.e. has been separated from the troglobitic lineages for more than 40 MY ago. All other major lineages, with Oligocene or Early Miocene origin, include only anophthalmous, endogean, interstitial or cave species (Additional file 1). This pattern strongly suggests that each of the geographical lineages diversified after the colonization of the subterranean medium took place, as suggested for other terrestrial [15,22,69,70] and stygobiontic [71] groups. This is in disagreement with both the climatic relict and the adaptive shift hypotheses, which assume multiple colonisations of the subterranean medium by closely related epigean ancestors and perceive the troglobitic taxa as evolutionary dead ends [18,20].

## Conclusions

We have shown here that the main lineages of Western Mediterranean Leptodirini have an origin in the Early-Mid Oligocene, and most likely developed all modifications to the subterranean life in the main geographical areas in which they are found today prior to the Late Oligocene-Miocene. The diversification within each of these main geographical areas seems thus to have taken place since early Miocene from ancestors fully adapted to the subterranean medium, contrary to most current assumptions about the evolution of the subterranean fauna.

## Authors' contributions

IR, AC and JF designed the study. JF and JMS obtained and identified the material. IR, RB, AI and AC did the molecular work and obtained the sequence data. IR did the phylogenetic analyses. IR and AC wrote a first draft, which final version was completed by IR, AC, APV, JF and JMS. All authors read and approved the final manuscript.

## Supplementary Material

Additional file 1 **Specimens used in the study, with locality, collector, voucher reference numbers and accession numbers for the sequences.** In grey, specimens combined in a composite sequence. Click here for file

Additional file 2 Estimation of the parameters for the Bayesian analyses. Click here for file

Additional file 3 **Cladogram obtained with MrBayes with the MAFFT alignment including the Sardinian species, with detailed node support values.** Upper row, MrBayes posterior probabilities, MF/PR; lower row, bootstrap support values (1,000 replicas) in Garli, MF/PR. Click here for file

Additional file 4 Phylogram obtained with MrBayes with the MAFFT alignment, with the exclusion of the Sardinian species. Click here for file

Additional file 5 **Ultrametric tree obtained with Beast using the *cox1 *sequence only, including the Sardinian species (red clade). **Black circles, well supported nodes (see Fig. 1, Additional file 3) constrained to be monophyletic. Numbers inside nodes, age estimate (MY) using the separation of the Sardinian species with a prior age of 33MY (see text). Click here for file

Additional file 6 **Age estimations of the nodes in Fig.**3**and Additional file**5. Vertical axis, estimated age (MY). Horizontal axis, nodes. White circles, estimation using the combined mitochondrial genes (*cox1*, *rrnL*+*trnL*, *nad1*, *cob*) in Beast, with 95% confidence intervals (dashed lines) (Fig. 3). Black circles, estimation using only *cox1 *in Beast, with 95% confidence interval (solid line) (Additional file 5). Red circles, estimation using the gen *cox1 *in r8s. Estimations using *cox1 *alone were calibrated with the node "Sardinian clade [cox1]", and those using the combined mtDNA with the node "Sardinian clade [mt]". Note than in the estimation using the combined mtDNA genes the Sardinian species were not included. Click here for file
