# Supplementary figures and images for: Ancient origin of a Western Mediterranean radiation of subterranean beetles
Source: BMC Evol Biol. 2010 Jan 28;10:29. doi: 10.1186/1471-2148-10-29 (PMC2834687; doi:10.1186/1471-2148-10-29)

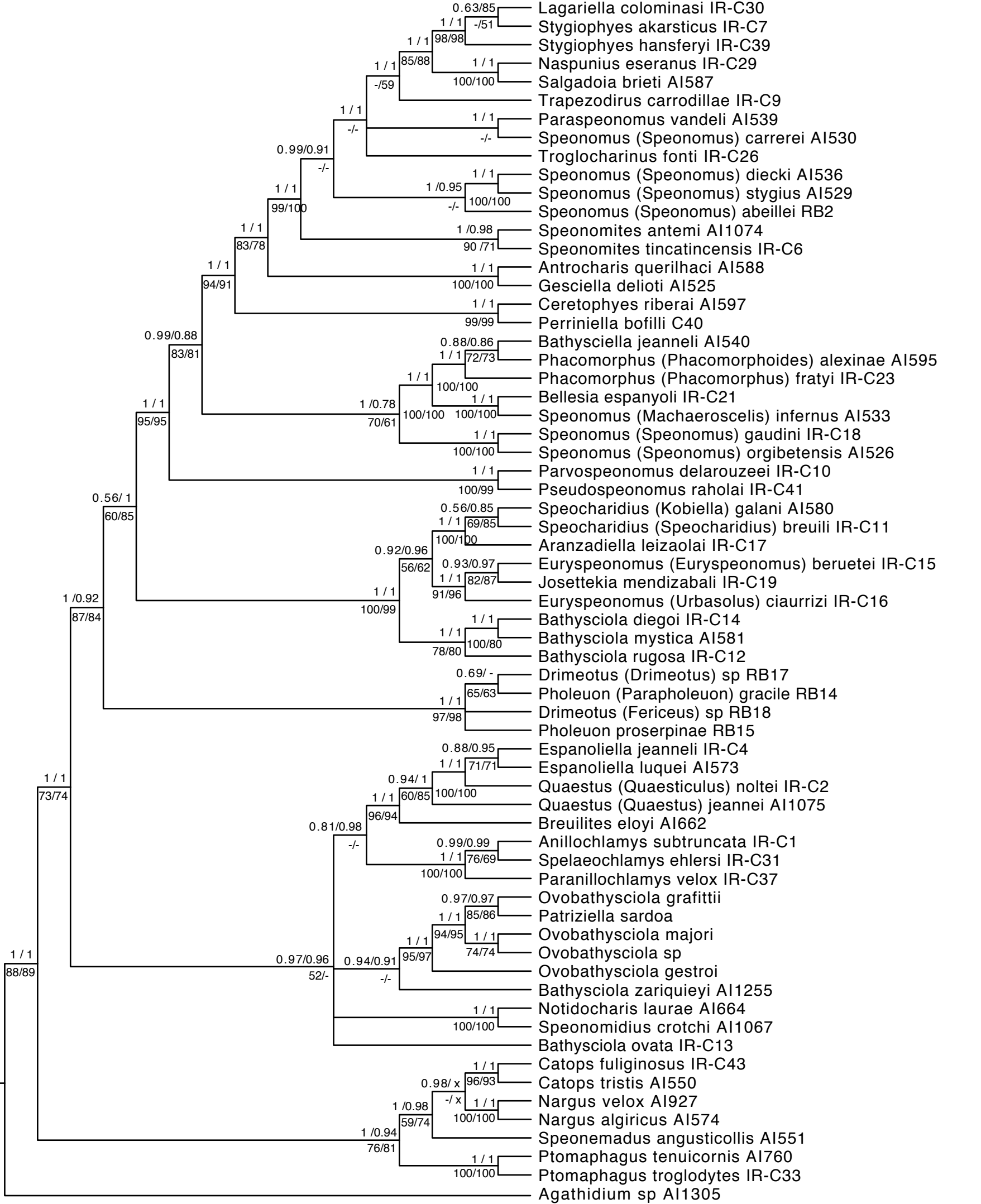

Supplement: Additional file 3 — Cladogram obtained with MrBayes with the MAFFT alignment including the Sardinian species, with detailed node support values. Upper row, MrBayes posterior probabilities, MF/PR; lower row, bootstrap support values (1,000 replicas) in Garli, MF/PR. [file 1471-2148-10-29-S3.PDF]

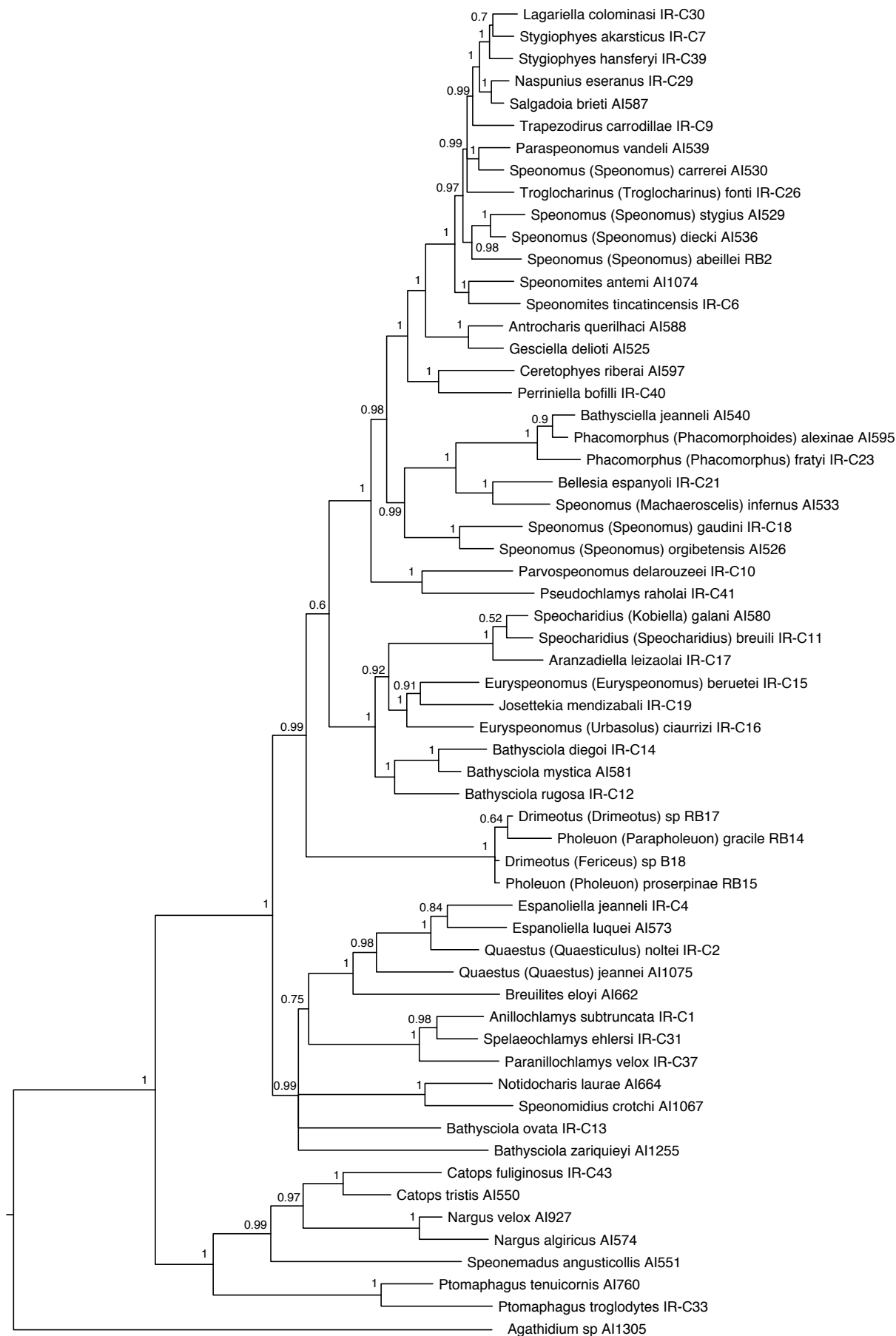

Supplement: Additional file 4 — Phylogram obtained with MrBayes with the MAFFT alignment, with the exclusion of the Sardinian species. [file 1471-2148-10-29-S4.PDF]

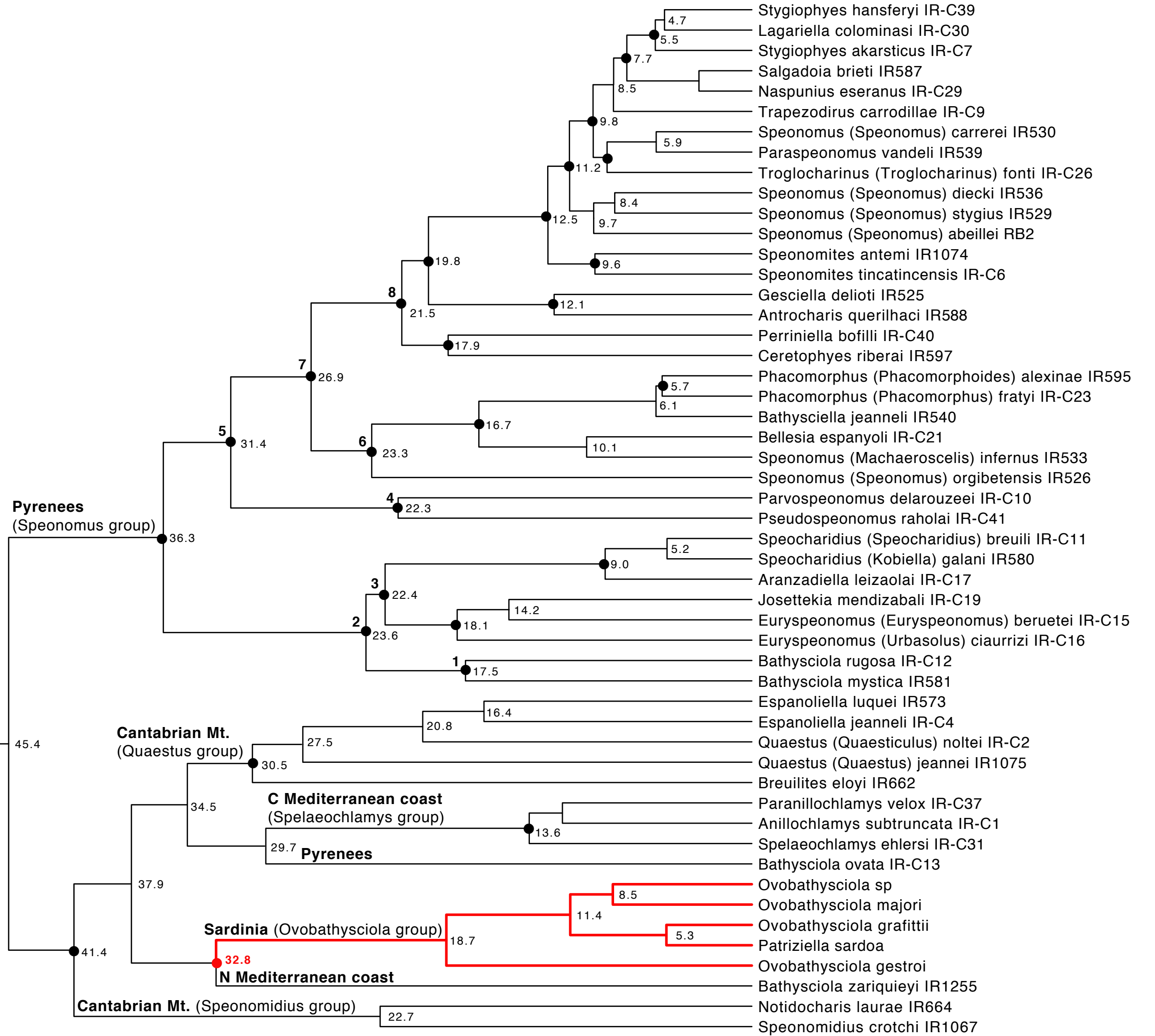

Supplement: Additional file 5 — Ultrametric tree obtained with Beast using the cox1 sequence only, including the Sardinian species (red clade). Black circles, well supported nodes (see Fig. 1, Additional file 3) constrained to be monophyletic. Numbers inside nodes, age estimate (MY) using the separation of the Sardinian species with a prior age of 33MY (see text). [file 1471-2148-10-29-S5.PDF]

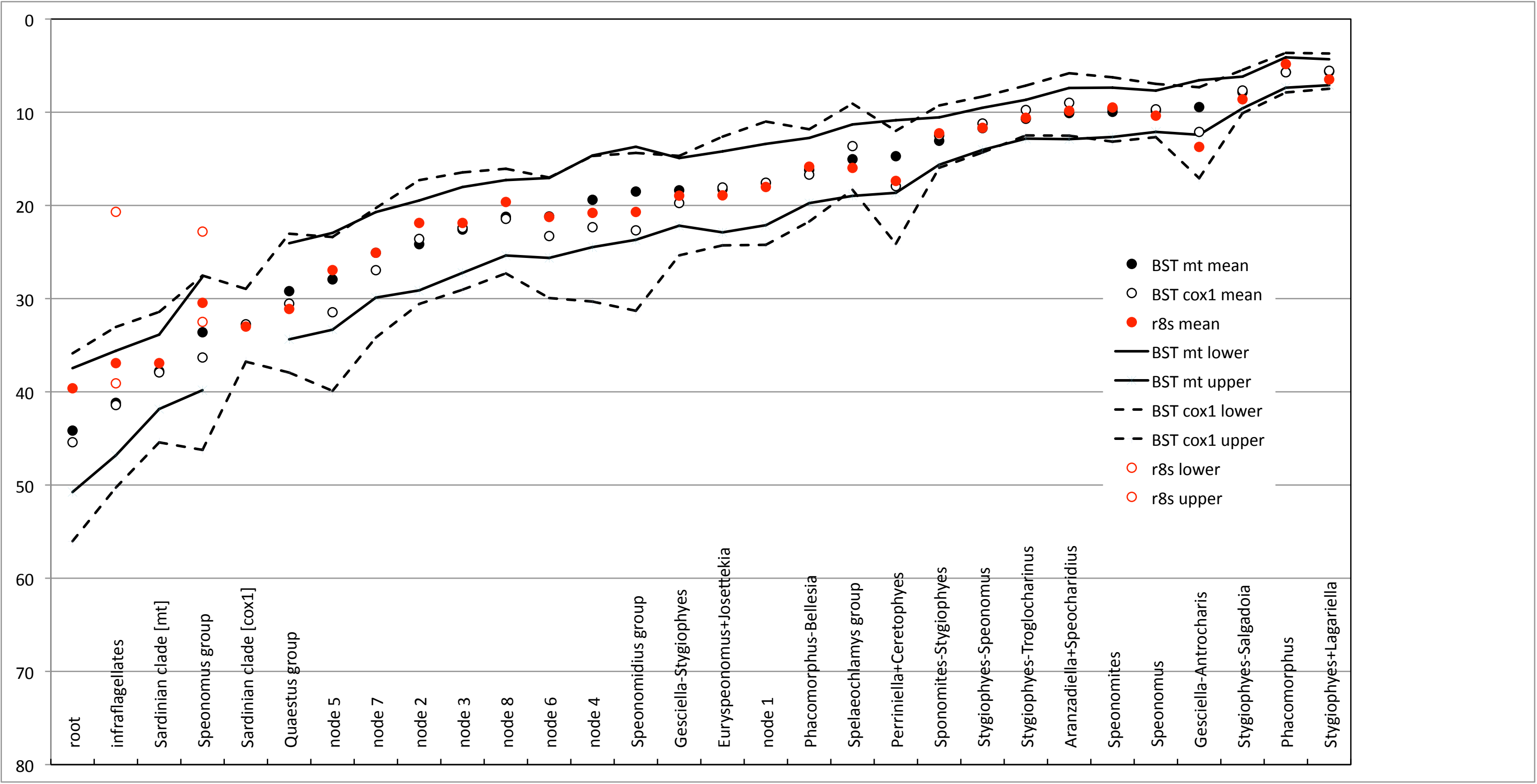

Supplement: Additional file 6 — Age estimations of the nodes in Fig.3and Additional file5. Vertical axis, estimated age (MY). Horizontal axis, nodes. White circles, estimation using the combined mitochondrial genes (cox1, rrnL+trnL, nad1, cob) in Beast, with 95% confidence intervals (dashed lines) (Fig. 3). Black circles, estimation using only cox1 in Beast, with 95% confidence interval (solid line) (Additional file 5). Red circles, estimation using the gen cox1 in r8s. Estimations using cox1 alone were calibrated with the node "Sardinian clade [cox1]", and those using the combined mtDNA with the node "Sardinian clade [mt]". Note than in the estimation using the combined mtDNA genes the Sardinian species were not included. [file 1471-2148-10-29-S6.PDF]
